# Response to letter to editor: ‘Comment on Arch et al., *Trials*. 2016;17:517’

**DOI:** 10.1186/s13063-017-1981-9

**Published:** 2017-05-26

**Authors:** B. N. Arch, J. Blair, A. McKay, J. W. Gregory, P. Newland, C. Gamble

**Affiliations:** 1 Department of Biostatistics, The University of Liverpool, Liverpool, L69 3BX UK; 2 Alder Hey Children’s NHS FT, East Prescott Road, Liverpool, L12 2AP UK; 3 Professor of Paediatric Endocrinology and Honorary Consultant, Division of Population Medicine, School of Medicine, Cardiff University, Heath Park, Cardiff, CF14 4XN UK; 4 Department of Biochemistry, Alder Hey Children’s NHS FT, East Prescott Road, Liverpool, L12 2AP UK

**Keywords:** HbA1c, Agreement, Trial design, Measurement

## Abstract

**Abstract:**

In October 2015 we published the paper ‘Measurement of HbA1c in multicentre diabetes trials – should blood samples be tested locally or sent to a central laboratory: an agreement analysis’. Chatterjee and Pradhan have submitted a letter to the editor asking critical questions regarding the methods we used.

We offer this letter in response.

**Trial registration:**

Eudract No. 2010-023792-25. Registered on 4 November 2010. ISRCTN No. ISRCTN29255275. Registered on 12 November 2010

## Main text

We thank Chatterjee and Pradhan for their letter regarding our paper in *Trials*. 2016; 17-517. We agree with their sentiment that local HbA1c measurement cannot be implemented at the expense of clinically unacceptable disparities between centralised and localised measurements despite its greater cost efficiency. We hope that the following provides the additional information that will aid their assessment of our results.

## Time-lag

In their letter, Chatterjee and Pradhan draw attention to Fig. 3 of our paper, and notice that within centres the distribution of differences is centred on 0 (suggesting that there are no centre-specific systematic biases present). We would argue that this does not imply that the same relationship would necessarily be true for time-lag. Figure 3 displayed results by site; however, within a site the time lag may vary. We have produced boxplots to show the distribution of differences by time-lag and a scatterplot as requested. This demonstrates an absence of a linear relationship between time-lag and discrepancy. They also indicate that in practice time-lag can be an important factor for high glucose values. As part of the underlying assumptions of the Bland-Altman method we investigated heteroscedasticity (see the *Verification of assumptions* section within the ‘Results’ section of our paper), i.e. we did not observe any increase in discrepancy with higher glycosylated haemoglobin (HbA1c).

**Fig. 1:**
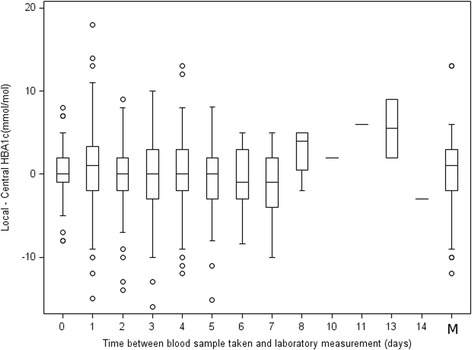
Boxplots showing the distribution of differences between local and central measurements by time-lag in days: between blood samples being taken and analysis at the central laboratory. M: measurements where the date of laboratory measurement was not recorded

**Fig. 2:**
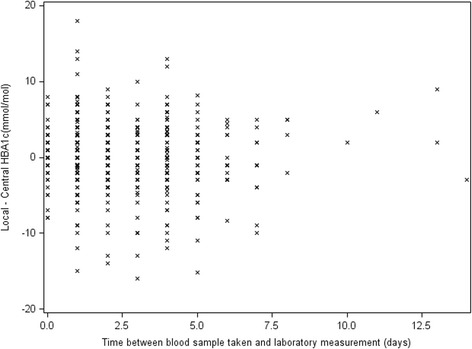
Scatterplot showing the distribution of the magnitude of differences between local and central measurements by time-lag in days: between blood samples being taken and analysis at the central laboratory

Of the 590 measurements analysed for agreement, for 79 (13.4%) the date of measurement at the central laboratory was not recorded. These 79 are indicated with ‘M’ on Fig. 1 and excluded from Fig. 2 (scatterplot). For the remaining 511, in only 8 (1.5%) measurements was there a time-lag of more than 7 days. The Pearson’s correlation between time-lag and difference in measurements (local minus central HbA1c) was found to be −0.02. This was statistically not different from 0 (*p* = 0.48). This means that there is no evidence of a straight-line relationship (linear correlation) between time-lag and agreement. (See Figs. 1 and 2)

## HbA1c measurement methodology

We specify in our paper that in almost all cases, both local and central, HbA1c was measured via immunoassay using portable machines. Local measurements were normally taken at outpatient clinics – but the technical method of measurement employed was not recorded. At the time of analysis, we contacted all sites to establish what the local methodology was. Table 1 gives details of what the sites’ responses were.

**Table 1:** Local HbA1c measurement methodology, as reported by sites

Centre Code	Local measurement methodology
1	Main method: DCA machine; 2 other possibilities: local laboratory or Hh9210 premier analyser machine by A Menarini Diagnostics
2	Machine in clinic
3	Diabetes team have their own machine
4	Method during follow-up: DCA machine in clinic calibrated daily with local laboratory
5	Machine on the ward
6	DCA Vantage in the Diabetes Centre. QC managed by pathology department in the hospital
7	Siemens DCA Vantage machine in clinic
8	Alfinion machine in outpatients
9	Portable DCA machine
10	Machine in clinic
11	Method during follow-up: DCA 2000 machine in clinic
12	Machine in clinic
13	Main method: local laboratory (till April 2015); then new analyser machine
14	Technician from local laboratory brings a machine to the clinic
15	DCA analyser for majority of follow-up appointments

*QC* quality control

**Fig. 3:**
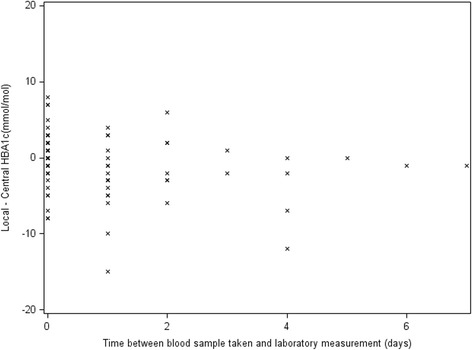
Scatterplot of discrepancies in measurements by time-lag at Alder Hey Hospital (location of central laboratory)

We can give further detail here to say that the same portable machine was used at Alder Hey for outpatient clinics as at the central laboratory (based at Alder Hey). At this centre therefore, the methodology was identical. Differences were still incurred despite using the same machine with a short time-lag and removing the courier and post-transfer issues (see Fig. 3).

Whether central laboratory results should be used in preference to local results is an issue that needs to be considered at the design stage of any study. We hope that the information presented will enable greater clarity in decisions made. However, any decision needs to be born against the size of the effect that is to be detected and the potential size of discrepancies. This study demonstrates that despite quality control placed on local machines such discrepancies do occur. It should also be emphasised that this study took place in the UK and the climate and transport conditions elsewhere may determine whether local measurements are preferable.

